# 

**Published:** 1892-08

**Authors:** 


VOL. XIII] *PUBLISHED MONTHLY AT THREE DOLLARS A YEAR. SINGLE COPIES, THIRTY CENTS.* [No. 8.

Entered at the Post Office, February 3rd, 1891, at New York, as Second-class Matter.
Copyright, 1892, by W. A Conklin and R. S. Huidekoper.
Printed for the Editors by E. SCOTT, 134 West 23d Street, New York.

LIBRARY Indexed S. G. O.

### THE JOURNAL OF COMPARATIVE MEDICINE AND VETERINARY ARCHIVES.

EDITED BY

W. A. CONKLIN, Ph. D., D. V S.

RUSH SHIPPEN HUIDEKOPER, M. D., Veterinarian, (Alfort),

*NEW YORK.*

AUGUST, 1892.

All communications and payments should be addressed to

**MANAGING EDITOR JOURNAL COMP. MEDICINE AND VET. ARCH.**

311 West 59th Street, New York.

**Copies may be had in London of BALLIERE, TINDALL & COX.**

